# Identification of a robust functional subpathway signature for pancreatic ductal adenocarcinoma by comprehensive and integrated analyses

**DOI:** 10.1186/s12964-020-0522-4

**Published:** 2020-03-02

**Authors:** Ping Wang, Chunlong Zhang, Weidong Li, Bo Zhai, Xian Jiang, Shiva Reddy, Hongchi Jiang, Xueying Sun

**Affiliations:** 1 The Hepatosplenic Surgery Center, the First Affiliated Hospital of Harbin Medical University, Harbin, 150001 China; 2 Department of Interventional Radiology, the Third Affiliated Hospital of Harbin Medical University, Harbin, 150086 China; 3 College of Bioinformatics Science and Technology, Harbin Medical University, Harbin, 150081 China; 4 Department of General Surgery, the Fourth Affiliated Hospital of Harbin Medical University, Harbin, 150001 China; 5 Department of Molecular Medicine & Pathology, Faculty of Medical and Health Sciences, the University of Auckland, Auckland, 1142 New Zealand

**Keywords:** Pancreatic ductal adenocarcinoma, Prognosis signature, Subpathway activity, Comprehensive analysis, Meta-analysis

## Abstract

**Background:**

Pancreatic ductal adenocarcinoma (PDAC) is a highly lethal malignancy and its mortality continues to rise globally. Because of its high heterogeneity and complex molecular landscapes, published gene signatures have demonstrated low specificity and robustness. Functional signatures containing a group of genes involved in similar biological functions may display a more robust performance.

**Methods:**

The present study was designed to excavate potential functional signatures for PDAC by analyzing maximal number of datasets extracted from available databases with a recently developed method of FAIME (Functional Analysis of Individual Microarray Expression) in a comprehensive and integrated way.

**Results:**

Eleven PDAC datasets were extracted from GEO, ICGC and TCGA databases. By systemically analyzing these datasets, we identified a robust functional signature of subpathway (path:00982_1), which belongs to the drug metabolism-cytochrome P450 pathway. The signature has displayed a more powerful and robust capacity in predicting prognosis, drug response and chemotherapeutic efficacy for PDAC, particularly for the classical subtype, in comparison with published gene signatures and clinically used TNM staging system. This signature was verified by meta-analyses and validated in available cell line and clinical datasets with chemotherapeutic efficacy.

**Conclusion:**

The present study has identified a novel functional PDAC signature, which has the potential to improve the current systems for predicting the prognosis and monitoring drug response, and to serve a linkage to therapeutic options for combating PDAC. However, the involvement of path:00982_1 subpathway in the metabolism of anti-PDAC chemotherapeutic drugs, particularly its biological interpretation, requires a further investigation.

**Video Abstract**

## Background

Pancreatic ductal adenocarcinoma (PDAC) is the fourth leading cause of cancer-related deaths worldwide and is predicted to be the second in the United States and Europe by 2030 [1, 2]. PDAC is regarded as a devastating malignancy due to its aggressive nature, presenting at an advanced stage and resistance to most treatment modalities, resulting in an overall 5-year survival rate at 9% [3], which is the lowest 5-year survival rate among all solid malignancies [4]. Such a poor outcome highlights an urgent need for seeking novel biomarkers to predict survival and monitor therapy response, which may also provide a more precise link to therapeutic options for combating PDAC.

PDAC has a very complex molecular landscape [5]. Efforts in deeply analyzing datasets have led to the discovery of potential PDAC gene signatures, which contain various numbers of distinguishable genes [6–16]. However, these reported signatures have few overlapping component genes with different functions, raising questions about their biological relevance, clinical significance and universal application for the management of PDAC. Each of them only reflects a specific biological trait because of cancer genetic instability, profusion of gene expression and diverse molecular subtyping, given that a high degree of heterogeneity among individuals and even within the same PDAC tumor [17, 18]. On the other hand, functions of genes and pathways explain the major features of pancreatic tumorigenesis and progression [19], thus functional signatures may display more robust performance since they contain a group of genes involved in similar biological functions [10, 20]. In order to excavate functional mechanism-anchored signatures, an analytical method called Functional Analysis of Individual Microarray Expression (FAIME) has been developed, which converts the transcriptomic information into molecular functional profiles [21]. By employing FAIME, we and others have identified several functional signatures for lung cancer [22], melanoma [23] and metabolic disorders [24]. We, therefore, designed the present study aiming at seeking potential functional signatures for PDAC by analyzing maximal number of datasets extracted from available public databases with FAIME in a comprehensive and integrated way.

## Materials and methods

### Datasets

Seven datasets extracted from databases of Gene Expression Omnibus (GEO) (https://www.ncbi.nlm.nih.gov/geo/) by using appropriate searching strategies (Supplementary Figure S1 and S2) were used as training sets, and 3 datasets from International Cancer Genome Consortium (ICGC) database (http://icgc.org/) and one from The Cancer Genome Atlas (TCGA) database (https://portal.gdc.cancer.gov/) were used as test sets (Supplementary Table S1).

Cell line datasets contained profiles of mRNA expression and drug sensitivity data of 44 and 32 human PDAC cell line samples were extracted from databases of Cancer Cell Line Encyclopedia (CCLE) and Genomics of Drug Sensitivity in Cancer (GDSC) up to March of 2019, respectively.

### Resources of pathways and subpathways

The pathway graphs were obtained from the Kyoto Encyclopedia of Genes and Genomes (KEGG) database [25] by using an R-based package called SubpathwayMiner [26] and converted into undirected graphs, where genes were represented by nodes. The subpathway graphs were defined based on the distance similarity rule [26] so that the distance of any two gene nodes was no larger than the cutoff *k* (default cutoff *k* = 3). Finally, a total of 300 pathways and 1773 subpathways were included in the study.

### Methods of comprehensive and integrated analyses

**Fig. 1:**
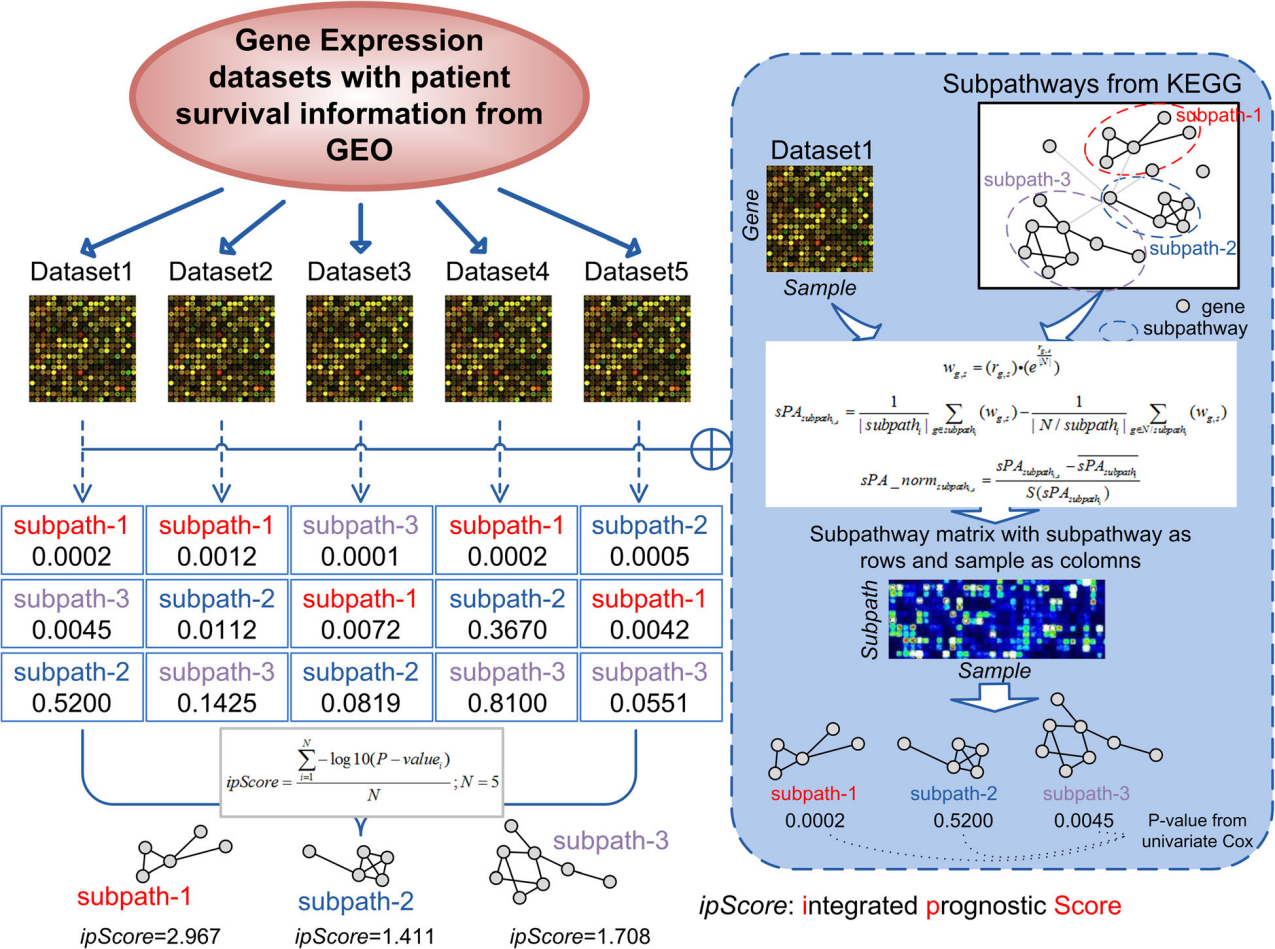
Outline of comprehensive and integrated analyses. An integrated prognostic score (ipScore) for each signature at gene, subpathway and pathway levels was calculated as described in Materials and Methods. An analysis at subpathway level by using a combination of 5 training sets is shown as an example

Comprehensive and integrated analyses were performed at levels of gene, subpathway and pathway by using various combinations of training sets, which consisted of 5, 6 or 7 training datasets. An example of the procedure for a combination of 5 datasets at the level of subpathway is shown in Fig. 1. The activities of each pathway and subpathway were evaluated by using a method of FAIME with modification [21].

Firstly, all the expressed genes (*N*_*g*_) from each sample were ranked in a descending order according to their expression levels, and the exponential decreasing weights (*w*) were calculated for the ordered genes (*w*_*g*, *s*_) by using Formula (1) as follows:

1 $$ {w}_{g,s}=\left({r}_{g,s}\right)\bullet \left({e}^{\frac{r_{g,s}}{\mid N\mid }}\right) $$

where *r*_*g*, *s*_ is the rank for gene *g* in sample *s*, and ∣*N*∣, the total number of genes in the sample. For analyzing the subpathway graph *i*, a component-set *subpath*_*i*_ indicates that it satisfies *component* ∈ *subpath*_*i*_ and *N*/*subpath*_*i*_, all the other components not included in the subpathway graph *i*. The score of subpathway *i* activity ($sP{A}_{subpat{h}_{i,s}}$) was calculated by using Formula (2) as follows:

2 $$ sP{A}_{subpat{h}_{i,s}}=\frac{1}{\mid subpat{h}_i\mid}\sum \limits_{g\in subpat{h}_i}\left({w}_{g,s}\right)-\frac{1}{\mid N/ subpat{h}_i\mid}\sum \limits_{g\in N/ subpat{h}_i}\left({w}_{g,s}\right) $$

The normalized score of subpathway activity ($sPA\_ nor{m}_{subpat{h}_{i,s}}$) was calculated by using Formula (3) as follows:

3 $$ sPA\_ nor{m}_{subpat{h}_{i,s}=}\frac{sP{A}_{subpat{h}_{i,s}}-\overline{sP{A}_{subpat{h}_i}}}{S\left( sP{A}_{subpat{h}_i}\right)} $$

where $\overline{sP{A}_{subpat{h}_i}}$ is the mean score of subpathway activity in all analyzed samples, and $S\left( sP{A}_{subpat{h}_i}\right)$, standard deviation.

The *P*-value of each subpathway in each training set was calculated by using a univariate Cox, and an integrated prognostic score (*ipScore*) of each subpathway in various combinations of training sets was calculated by using Formula (4) as follows:

4 $$ ipScore=\frac{\sum \limits_i^N-\log 10\left(P- valu{e}_i\right)}{N} $$

where N (=5, 6 or 7) is the number of training sets in combinations.

The *ipScore* for each pathway was calculated and normalized to form the pathway activity matrix by using the same method as described above. And for gene level analysis, a univariate cox was performed based on the gene expression level, and the *ipScore* for each gene was calculated according to the Formula (4).

### Meta-analyses

The software STATA (version 14) was employed to evaluate hazard ratio (HR), and a funnel plot, the publication bias. Heterogeneity was assessed by using the I^2^ statistic according to the Cochrane handbook for systematic reviews of interventions and *I*^2^ > 50% indicates the existence of substantial heterogeneity. A fixed-effect model was used to summarize the results when *I*^2^ < 50%, otherwise, a random-effect model was used. The possible source of heterogeneity was evaluated by sensitive analysis.

### Statistical analyses

A univariate Cox method was employed to evaluate the correlation between the signature and the survival. A K-mean clustering method (K = 2) was performed for analyzing multiple variable signatures. A log-rank test was used to compare the difference in survival between the two groups. Statistical analyses for hierarchical cluster, spearman correlation, hypergeometric test, wilcoxon rank sum test and Cox proportional hazards were performed by using an R software package (Version 3.1.0). *P*-value < 0.05 is considered statistically significant.

## Results

### Excavation of gene, subpathway and pathway signatures

**Fig. 2:**
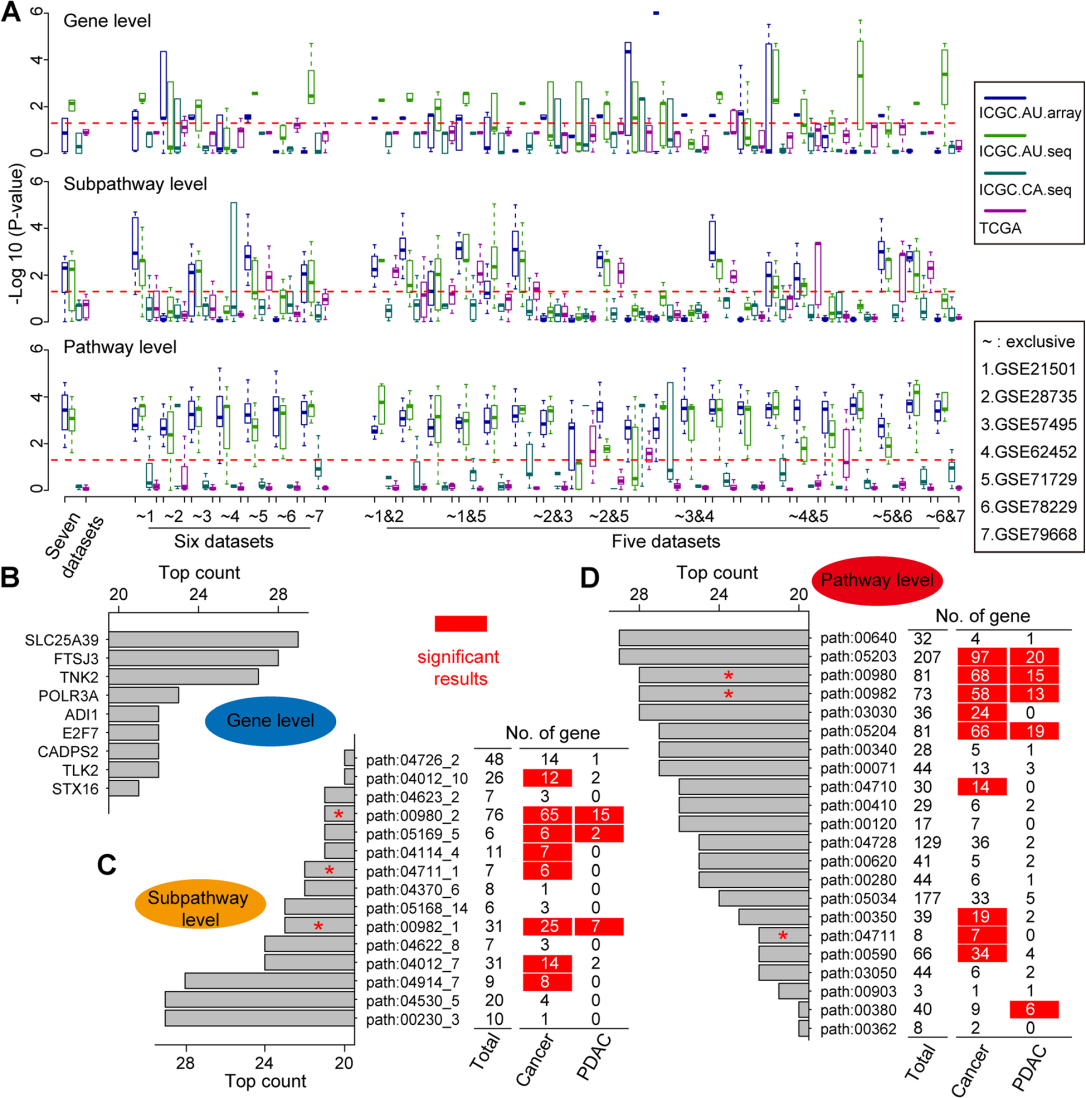
Excavation of potential signatures associated with PDAC. **a** The integrated prognostic score for each signature is calculated and assessed as described in Materials and Methods. *P*-value is calculated and -log10 of *P*-value used as Y-axis. **b-d** Top-counted signatures identified at levels of gene (**b**), subpathway (**c**) and pathway (**d**). Percentages of cancer- and PDAC-related genes are calculated and *P*-values, determined by a hypergeometric test in (**c**) and (**d**). Significant results are marked by red color. “*” indicates that the pathway or subpathway was selected for further analyses

An *ipScore* of each biomarker was calculated in 29 different combinations (each contained 5, 6 or 7 datasets) of the 7 training sets. Based on their ranks, top 30 signatures obtained from each combination were regrouped into serial 28 sets, which contained 3–30 signatures (1st-3rd, 1st-4th, 1st-5th … ...1st-30th), respectively, and were further assessed in the 4 test sets (Fig. 2a). A high heterogeneity existed among different combinations, but functional signatures showed a higher robustness than gene signatures (Fig. 2a).

The appearing frequency of each signature from each combination was counted in the other 28 combinations at levels of gene, subpathway and pathway (Analytical data File 1–3**)**. Top counted signatures showed cumulative effects as assessed in the test sets, the predictive capacity became more robust when the number of signatures was ≥10 (Supplementary Figure S3).

Based on their appearing frequency (cut-off ≥20) in all the 29 combinations, 9 genes (Fig. 2b), 15 subpathways (Fig. 2c) and 22 pathways (Fig. 2d) were selected as candidate signatures, whose relevance with PDAC was further examined by using datasets of 3000 cancer-related and 250 PDAC-related genes derived from Genetic Association Database (GAD). None of 9 gene signatures are PDAC-related (Fig. 2b), in accordance with their poor predictive ability (Fig. 2a). By analyzing the biological functions, commonalities, intersection points and their subsidiary relationship, we chose three pairs (path:00980_2/path:00980, path:00982_1/path:00982 and path:00477_1/path:00477) for further analyses because they were shown to be associated with cancer and/or PDAC at both pathway and subpathway levels (Fig. 2c and d).

### Identification of the path:00982_1 subpathway signature

**Fig. 3:**
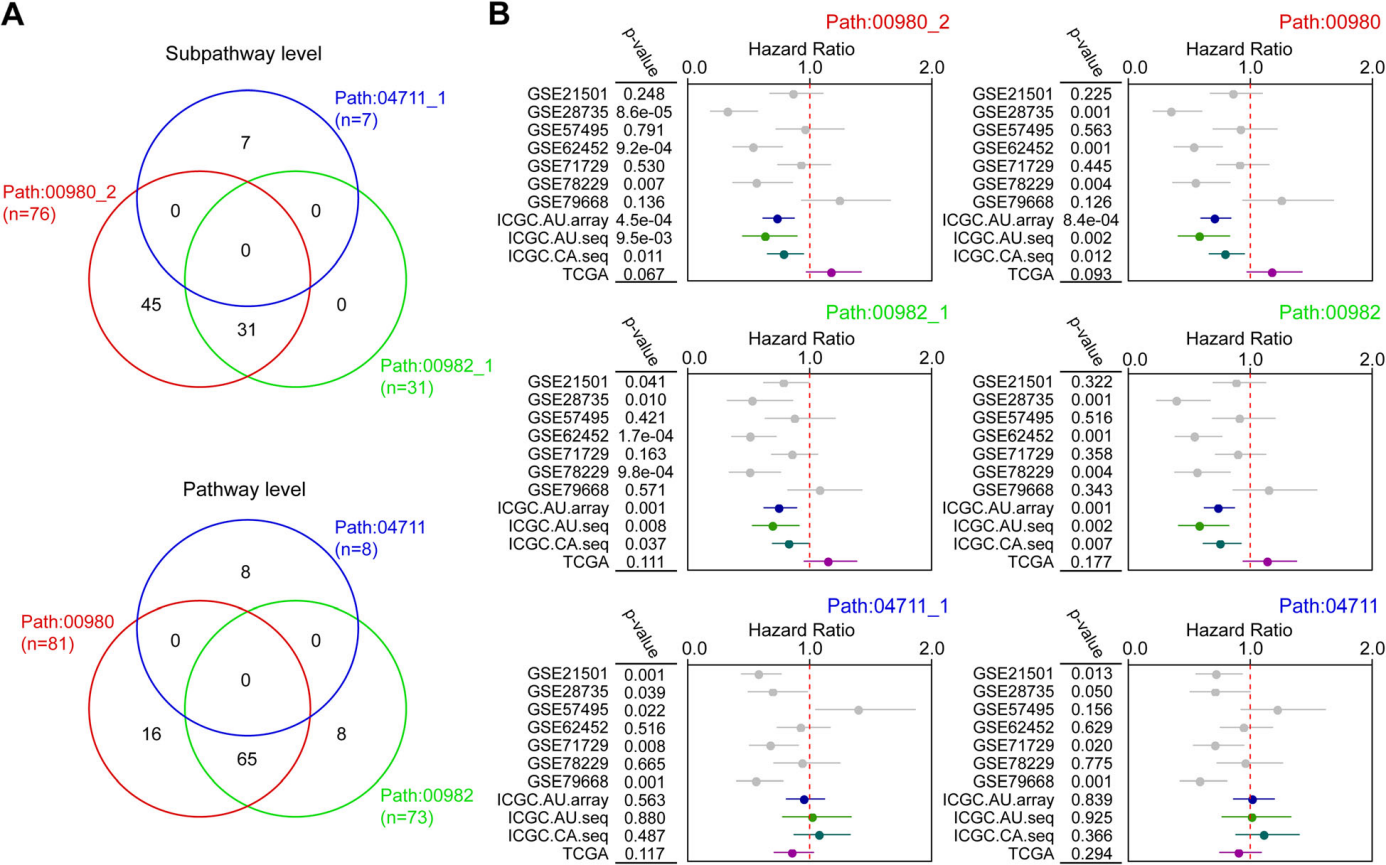
Analysis of the overlapping relationship and predictive ability of signatures. **a** Overlapping genes of pathways and subpathways. “n” indicates the number of genes. **b** The predictive ability of signatures in the 11 datasets. Hazard ratio and *P*-value are calculated by using a univariate Cox analysis

We next analyzed whether these three pairs shared common genes at the levels of subpathway and pathway. As shown in Fig. 3a, the path:00980_2 subpathway covered all the genes of path:00982_1 subpathway, while path:00980 pathway covered most genes (65/73, 89.04%) of path:00982 pathway; but neither path:00477_1 subpathway nor path:04711 pathway shared any genes with the other two pairs. The prognostic capacity of each signature was analyzed in each dataset by using a univariate Cox analysis, which showed that the path:00980_2/path:00980 and path:00982_1/path:00982 signatures had higher predictive capacities than path:00477_1/path:00477 signatures (Fig. 3b). Based on the above comprehensive and integrated analyses and considering the number of genes and the overall predictive capacity, we finally selected the path:00982_1 signature (Supplementary Figure S4 and Supplementary Figure S2) for further analysis.

### The path:00982_1 signature is a protective signature for PDAC

**Fig. 4:**
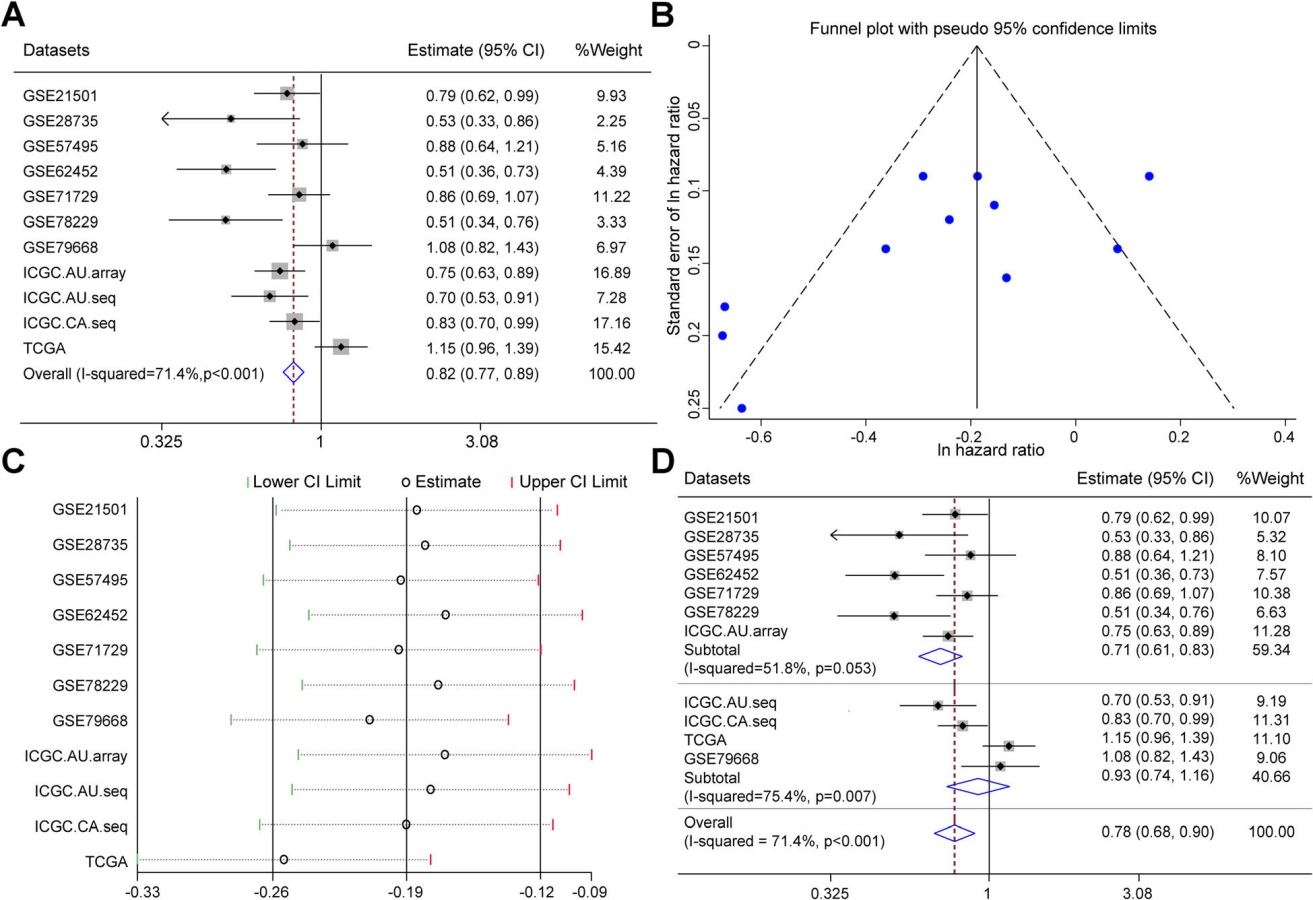
Meta-analysis of the predictive capacity of path:00982_1 signature. **a** Forest plots of pooled hazard ratio for analyzing the impact of path:00982_1 signature on the survival in each dataset. **b** Funnel plots of meta-analysis. **c** Sensitivity analysis of meta-analysis. **d** Forest plots for analyzing the impact of path:00982_1 signature in two subgroups, microarray (the upper panel) and RNA sequencing (the middle panel). I-square and *P*-value in each subgroup (subtotal) and for all datasets (overall, the lower panel) are calculated. ES, estimates; CI, confidence interval

Meta-analyses showed that the path:00982_1 signature was a significantly protective factor for PDAC with an overall pooled HR of 0.82 (95% confidence interval [CI] 0.77, 0.89; *p* < 0.001) (Fig. 4a). The funnel was generally symmetrical without obvious publication biases, indicating the results of meta-analyses were credible (Fig. 4b). However, the heterogeneity (*I*^2^ = 71.4%) was high as analyzed by using sensitivity analyses. The overall pooled estimate could be reduced by excluding GSE79668 and TCGA datasets, and in particular, exclusion of TCGA dataset made the overall pooled estimate even closer to the lower CI limit (Fig. 4c). After further investigating the detailed techniques employed for generating gene expression profiles of each dataset, we found that RNA-seq (RNA sequencing) techniques were used in GSE79668 and TCGA datasets. We thus classified all the datasets into microarray and RNA-seq subgroups. By using meta-analyses, we found that the microarray subgroup had an overall pooled HR of 0.71 (95% CI 0.61, 0.83; *p* = 0.053) and an I^2^ of 51.8%, indicating a low heterogeneity; however, the RNA-seq subgroup had an overall pooled HR of 0.93 (95% CI 0.74, 1.16; *p* = 0.007) and an I^2^ of 75.4%, indicating a high heterogeneity in this subgroup (Fig. 4d). Based on the above results, we postulate that the heterogeneity is caused by RNA-seq techniques.

In addition, by using multivariate Cox analyses, we found that the path:00982_1 signature was an independent predictive factor as examined in all the available datasets, which included clinical information of age, gender, ethnicity, lymph nodes, grade, maximum tumor dimension, TNM (tumor, lymph nodes & metastasis) stages, N classification, molecular subtype (classical and basal) and history of diabetes [27–29] (Analytical data File 4).

### The path:00982_1 signature displays a higher prognostic capacity for the classical subtype

**Fig. 5:**
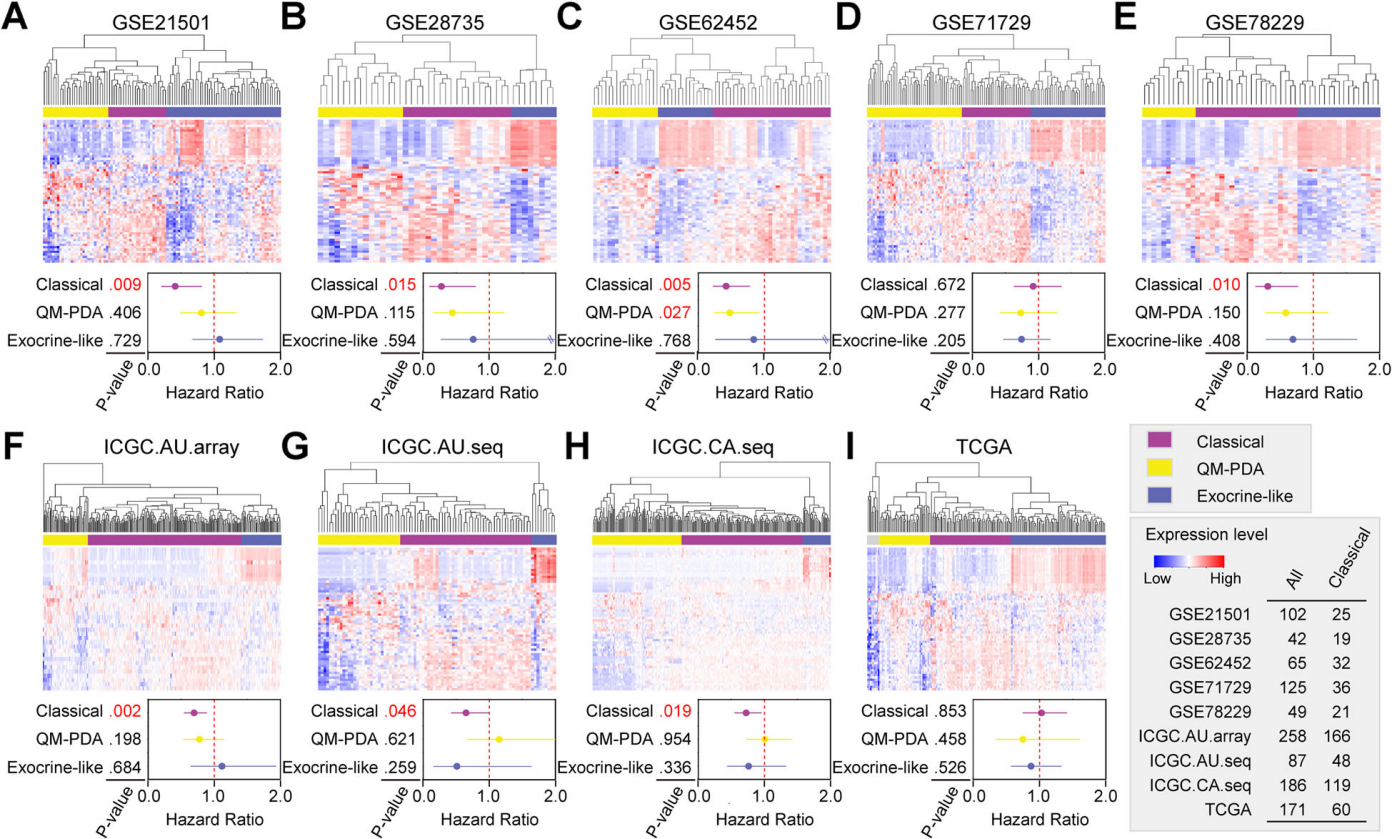
The prognostic capacity of path:00982_1 signature in different PDAC subtypes. PDAC patients in each dataset are stratified into three subtypes: Classical, Quasi-mesenchymal (QM-PDA) and Exocrine-like, with the classification [30]. Hazard ratio and *P*-value for each subtype are calculated. Data from 5 training datasets and 4 testing datasets are shown in the upper and lower panels, respectively. A number in red color indicates a significance

By adopting a published classification [30], we stratified PDAC patients into classical, quasi-mesenchymal (QM-PDA) and exocrine-like subtypes. Except for GSE57495 and GSE79668 datasets (Supplementary Figure S5), PDAC patients could be classified into three subtypes in the other 9 datasets (Fig. 5). The path:00982_1 signature demonstrated a significant predictive capacity for the classical subtype in 7 datasets (exclusive of GSE71729 and TCGA) (Fig. 5). By using another classification [31], we stratified PDAC patients into classical, basal-like and “others” subtypes. The path:00982_1 signature demonstrated a significant predictive capacity for the classical subtype in GSE21501, GSE28735, GSE62452 and ICGC.CA.seq datasets (Supplementary Figure S6).

### The path:00982_1 signature appears to be associated with the efficacy of chemotherapy for PDAC

**Fig. 6:**
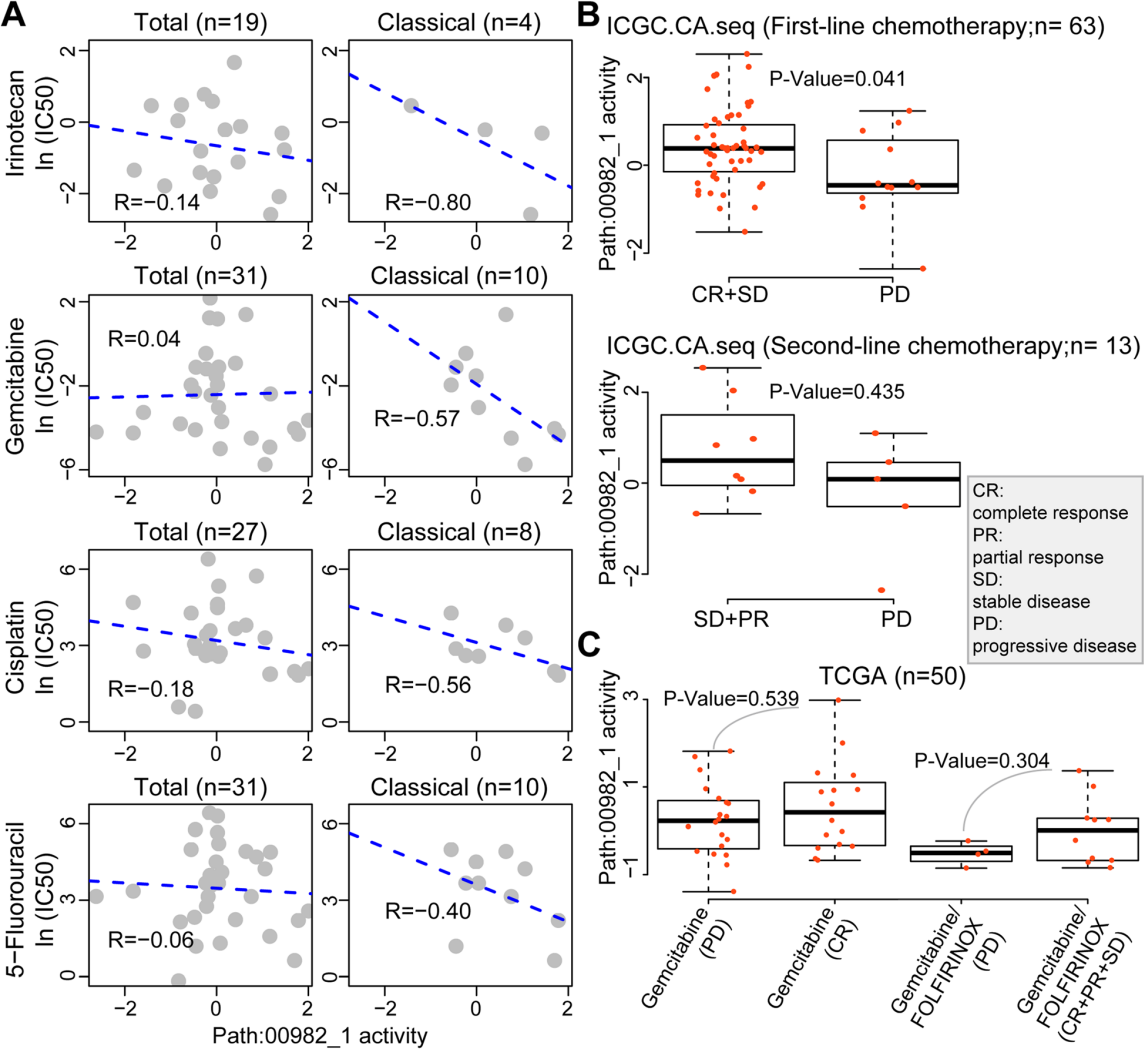
Correlation of path:00982_1 signature and chemotherapeutic effects. **a** Correlation of path:00982_1 activity and the IC_50_ of each chemotherapeutic drug against PDAC cell lines derived from CCLE and GDSC databases. *R* value is calculated by using a Spearman method. **b**, **c** Correlation of path:00982_1 activity and chemotherapeutic efficacy in the ICGC.CA.Seq (**b**) and TCGA (**c**) databases. Tumor responses are classified into complete response (CR), partial response (PR), stable disease (SD) and progressive disease (PD). *P*-value is calculated by using a Wilcoxon rank sum test. “n” in brackets refers to the number of patients. “Classical” indicates “classical subtype” according to the classification [30]

The path:00982_1 subpathway belongs to the drug metabolism-cytochrome P450 (CYP) pathway, which is responsible for drug response and the survival of PDAC patients [32–34]. We therefore explored its intervention with anti-PDAC drugs contained in the standard chemotherapeutic regimens FOLFIRINOX (folinic acid-fluorouracil-irinotecan-oxaliplatin) and gemcitabine plus nab-paclitaxel [35, 36]. Since the data of oxaliplatin and nab-paclitaxel were unavailable, we were only able to analyze the data of half maximal inhibitory concentration (IC_50_) of irinotecan, gemcitabine, cisplatin (belonging to platinum-based drugs as oxaliplatin) and 5-fluorouracil in PDAC cell lines derived from CCLE and GDSC databases (Analytical data File 5 and 6). A negative correlation was found between the IC_50_ of each drug and the activity of path:00982_1 subpathway in PDAC cells of classical subtype though it was moderate possibly because of small number of samples (Fig. 6a). We next employed a permutation analysis, in which the same number of samples of classical subtype were randomly selected from total samples, and the correlation between the path:00982_1 activity and the IC_50_ of each drug was calculated for 10,000 times. The number of times (N) was counted when the correlation value was less than the real correlation value, and *P*-value was calculated by using a formula (N/10000). The results indicated that the real correlation for irinotecan and gemcitabine was significant (*P* = 0.0248 and *P* = 0.0265, respectively) but not for cisplatin or 5-fluorouracil (*P* = 0.1546 and *P* = 0.0934, respectively) in PDAC cells of classical subtype.

We next searched for available clinical chemotherapy data in all the datasets and were only able to extract 63 and 50 cases from the ICGC.CA.seq and TCGA datasets, respectively. Patients were classified into subgroups depending on tumor responses, complete response (CR), partial response (PR), stable disease (SD) and progressive disease (PD). As shown in Fig. 6b, the path:00982_1 activity in CR + SD subgroups was significantly higher than that in PD subgroup extracted from ICGC.CA.seq dataset, in which patients received the first-line chemotherapy. The path:00982_1 activity was slightly higher in SD + PR subgroups than PD subgroup, in which patients received the second-line chemotherapy, but the difference did not reach significance (Fig. 6b). Among cases extracted from TCGA dataset, the difference in path:00982_1 activity between PD and CR subgroups receiving gemcitabine or between PD and CR + PR + SD subgroups receiving gemcitabine/FOLFIRINOX was not significant (Fig. 6c). Because the number of samples that contained intact data was too small, we were unable to stratify these patients into molecular subtypes for further analysis.

## Discussion

Here we report a functional path:00982_1 subpathway signature, which displays a robust and significant capacity in predicting survival, drug response and chemotherapeutic efficacy of PDAC, particularly those of classical subtype, accounting for 48.5% of all PDAC subtypes (Fig. 5). To our knowledge, this may be the first functional signature identified from a systematic study of the largest number of PDAC datasets involving comprehensive and integrated analyses with FAIME.

The TNM staging system is a globally recognized standard for classifying the extent of spread of cancer and is widely accepted for predicting the prognosis and guiding treatment options for PDAC. The N classification of the 8th Edition of the American Joint Committee on Cancer (AJCC) scheme for PDAC, particularly recently proposed LNR (lymph node ratio)-based N classification for respectable PDAC, has been shown to more accurately predict patient response [27–29]. Therefore, we employed a multivariate Cox analysis to compare the predicting power of our signature with this clinical system. As shown in Analytical data File 4, only N classification in GSE21501 dataset, TNM staging in GSE57495 dataset and number of positive lymph nodes in TCGA dataset were significantly correlated with the prognosis. The results indicate that this clinical system needs further optimization in predicting the prognosis of PDAC, in accordance with a previous study [30]. In comparison, the path:00982_1 signature displayed a significant predictive capacity in 5 datasets (*P* < 0.05) and a marginally significant predictive ability in 2 datasets (0.1 < *P* > 0.05) in this analysis (Analytical data File 4).

Until now, 11 studies on the identification of gene signatures in PDAC have been published [6–16]. By using multivariate Cox methods, we retrospectively analyzed the predictive capacity of these signatures in the present 11 datasets, in comparison with the path:00982_1 signature. The results showed that the path:00982_1 signature was more robust than any of the published gene signatures (Supplementary Table S3). For instance, the most powerful signature reported by Haider, et al. [8] among all the published gene signatures was shown to be significant in 6 datasets, while the path:00982_1 signature was significant in 7 datasets. In addition, our study has used 7 training sets and 4 test sets, while maximal 4 datasets including only one test set were used in any of the above 11 published studies. More advantageously the path:00982_1 signature was verified for different PDAC molecular subtypes and further validated in cell line and clinical datasets with chemotherapeutic efficacy [35, 36].

Chemotherapy plays an important role in the management of PDAC because of its aggressive nature and being diagnosed at an advanced stage [36, 37]. The path:00982_1 subpathway is located at the downstream of CYPs (Supplementary Figure S7), which constitute a large enzyme family that account for about 75% of the total drug metabolism [38]. Therefore, we analyzed the correlation of clinically used anti-PDAC chemotherapeutic drugs and the activity of path:00982_1 subpathway in available cell line and clinical datasets. The correlation between IC_50_ of each drug (irinotecan, gemcitabine, cisplatin and 5-fluorouracil) and path:00982_1 activity was only moderate though the correlation was higher in classical subtype than in the overall samples. To further analyze the data, we adopted a permutation analysis, which confirmed that the real correlation for irinotecan and gemcitabine was significant but not for cisplatin or 5-fluorouracil in PDAC cells of classical subtype. We next analyzed the path:00982_1 activity in PDAC patients, who were classified based on tumor response to chemotherapy. A significant result was found in patients receiving the first-line chemotherapy but not the second-line chemotherapy in the ICGC.CA.seq dataset, and not in TCGA dataset as well. Because that the path:00982_1 subpathway is composed of four groups of enzymes (Supplementary Table S2) [25], we further studied these enzymes and tried to seek the association with the above chemotherapeutic drugs. CYP2A6 participates in the metabolism of fluorouracil and CYP3A4 is involved with irinotecan pharmacokinetics [39], CYP2A6 is associated with the efficacy of SOX (S-1 plus oxaliplatin) regimen [40], and CYP4F2 partakes in the metabolism of gemcitabine [41]. However, majority of molecules in this subpathway are unable individually to exhibit a significant predictive ability (Analytical Data File 7) by using a univariate Cox method to evaluate the correlation between each gene and the survival of PDAC patients. The unexpected results may imply that this functional signature should be treated as an integrated enzyme complex, in which the 31 enzymes interact each other and work jointly to generate a biological function. The present results also emphasize the necessity of exploring functional signatures, rather than individual genes for PDAC with a high degree of heterogeneity [17, 18]. However, the role of path:00982_1 subpathway in the metabolism of anti-PDAC chemotherapeutic drugs, particularly its biological interpretation, requires further investigation.

The present study has several limitations, which need to be coped in the future. One is that the identified signature has not been verified in low-throughput experiments and the key nodes involved in this subpathway signature needs to be further mined. Another limitation is that the patients were not stratified into PDAC subtypes due to the small number of samples in available clinical datasets that contained intact profiles of chemotherapy efficacy, survival and gene expression, which may be the reason why the correlation between path:00982_1 activity and tumor response to chemotherapy was not shown to be significant. Finally, the predictive ability of this signature was not exhibited in 4 out of 11 datasets possibly because of a high inter-study heterogeneity resulting from the sample processing, diverse molecular subtyping and particularly RNA-seq techniques. The results also suggest that FAIME may not be suitable for analyzing those datasets when the gene expression profiles were generated by using RNA-seq techniques possibly because that FAIME was developed for microarray expression profiles.

## Conclusion

In summary, the present study has identified a novel robust functional signature, which displays a more powerful capacity in predicting the survival and chemotherapy response for patients with PDAC of classical subtype than the published gene signatures and the TNM staging system. This discovery may have an impact to some extent on clinical PDAC practice in the future in three aspects. Firstly, PDAC patients, particularly those of classical subtype, could be selected based on the activity of this subpathway so that chemotherapeutic regimens would be precisely and effectively targeted to those with higher path:00982_1 subpathway activity. Secondly, the signature could be used to improve the current systems for predicting the prognosis and monitoring drug response. Finally, interventions that increase the activity of this subpathway may be applied together with anti-PDAC drugs so that the efficacy of current chemotherapy may be improved. However, the present study has several limitations as mentioned above, and the involvement of path:00982_1 subpathway in the metabolism of anti-PDAC chemotherapeutic drugs, particularly its biological interpretation, requires further investigation.

## Supplementary information

**Additional file 1: Table S1.** Descriptive summary of datasets used in the study. **Table S2.** Genes of the 00982_1 subpathway. **Table S3.** Predictive power of published gene signatures. **Figure S1.** Dataset Search strategy in GEO database. **Figure S2.** Flow diagram of dataset selection strategies. **Figure S3.** Accumulative predictive abilities of signatures. **Figure S4.** Genes in the path:00982_1 subpathway. **Figure S5.** Collision classification for GSE57495 and GSE79668 datasets. **Figure S6.** Prognostic capacity of path:00982_1 signature for classical subtype. by Moffitt classification. **Figure S7.** Association of path:00982_1 subpathway with other pathways.

## Data Availability

The data generated are included in the manuscript and supplementary data. Analytical data including 7 files (Named File 1–7) are available at Mendeley Data (DOI: 10.17632/987jp9w76f.1).

## Abbreviations

CCLE: Cancer Cell Line Encyclopedia
FAIME: Functional Analysis of Individual Microarray Expression
GAD: Genetic Association Database
GDSC: Genomics of Drug Sensitivity in Cancer
GEO: Gene Expression Omnibus
ICGC: International Cancer Genome Consortium
KEGG: Kyoto Encyclopedia of Genes and Genomes
PDAC: Pancreatic ductal adenocarcinoma
TCGA: The Cancer Genome Atlas
