# Supplementary material for: Identification of a robust functional subpathway signature for pancreatic ductal adenocarcinoma by comprehensive and integrated analyses
Source: Cell Commun Signal. 2020 Mar 2;18:34. doi: 10.1186/s12964-020-0522-4 (PMC7053133; doi:10.1186/s12964-020-0522-4)
Supplement: Supplementary file 2 — Additional file 1: Table S1. Descriptive summary of datasets used in the study. Table S2. Genes of the 00982_1 subpathway. Table S3. Predictive power of published gene signatures. Figure S1. Dataset Search strategy in GEO database. Figure S2. Flow diagram of dataset selection strategies. Figure S3. Accumulative predictive abilities of signatures. Figure S4. Genes in the path:00982_1 subpathway. Figure S5. Collision classification for GSE57495 and GSE79668 datasets. Figure S6. Prognostic capacity of path:00982_1 signature for classical subtype. by Moffitt classification. Figure S7. Association of path:00982_1 subpathway with other pathways. [file 12964_2020_522_MOESM1_ESM.doc]

**Supplementary Data**

**Identification of a novel robust functional subpathway signature for pancreatic ductal adenocarcinoma by comprehensive and integrated analyses**

Ping Wang ^1,2^, Chunlong Zhang^3^, Weidong Li ^1,4^, Bo Zhai^1,4^, Xian Jiang^1^, Shiva Reddy^5^, Hongchi Jiang^1^, Xueying Sun^1^,*

^1^ The Hepatosplenic Surgery Center, the First Affiliated Hospital of Harbin Medical University, Harbin 150001, China

^2^ Department of Interventional Radiology, the Third Affiliated Hospital of Harbin Medical University, Harbin 150086, China

^3^ College of Bioinformatics Science and Technology, Harbin Medical University, Harbin 150081, China

^4^ Department of General Surgery, the Fourth Affiliated Hospital of Harbin Medical University, Harbin 150001, China

^5^ Department of Molecular Medicine & Pathology, Faculty of Medical and Health Sciences, the University of Auckland, Auckland 1142, New Zealand

**Contents**

**Original analytical data:** These data including 7 files (Named File 1-7) can be found at Mendeley Data (DOI: 10.17632/987jp9w76f.1).

**Supplementary Tables** .................................................................................................3

**Table S1.** Descriptive summary of datasets used in the study………….......................3

**Table S2.** Genes of the 00982_1 subpathway…………………………….…….……..4

**Table S3.** Predictive power of published gene signatures……….……..………..........5

**Supplementary Figures……………………………………………………...**...…….6

**Figure S1.** Dataset Search strategy in GEO database…………………………….......6

**Figure S2.** Flow diagram of dataset selection strategies..............................................7

**Figure S3.** Accumulative predictive abilities of signatures..........................................8

**Figure S4.** Genes in the path:00982_1 subpathway......................................................8

**Figure S5.** Collision classification for GSE57495 and GSE79668 datasets………....9

**Figure S6.** Prognostic capacity of path:00982_1 signature for classical subtype

by Moffitt classification...............................................................................9

**Figure S7.** Association of path:00982_1 subpathway with other pathways…….….10

**References**..................................................................................................................11

**Supplementary Tables**

**Table S1.** Descriptive summary of datasets used in the study

| **Dataset Name** | **GSE21501** | **GSE28735** | **GSE57495** | **GSE62452** | **GSE71729** | **GSE78229** | **GSE79668** | **ICGC.AU** | **ICGC.AU** | **ICGC.CA** | **TCGA** |
| --- | --- | --- | --- | --- | --- | --- | --- | --- | --- | --- | --- |
| **Platform** | GPL4133^a^ | GPL6244^b^ | GPL15048^c^ | GPL6244^b^ | GPL20769^d^ | GPL6244^b^ | GPL11154^e^ | NM^f^ | NM^g^ | NM^g^ | NM^e^ |
| No. of genes | 19751 | 20185 | 22584 | 20185 | 15667 | 20185 | 16973 | 13389 | 18942 | 18480 | 16912 |
| **Patients and phenotypes** |  |  |  |  |  |  |  |  |  |  |  |
| No. of Samples  (with survival data) | 132  (102) | 90  (42) | 63  (63) | 130  (65) | 357  (125) | 50  (49) | 51  (51) | 269  (258) | 91  (87) | 234  (186) | 177  (171) |
| Gender |  |  |  |  |  |  |  |  |  |  |  |
| Male | - | - | - | - | - | - | 32 | 138 | 45 | 102 | 93 |
| Female | - | - | - | - | - | - | 19 | 120 | 42 | 84 | 78 |
| Stages |  |  |  |  |  |  |  |  |  |  |  |
| I (Ia, Ib) | 1,6 | - | 0,12 | 0,4 | - | 0,4 | 2,7 | - | - | 11,41 | 5,15 |
| II (IIa, IIb) | 19,70 | - | 17,33 | 10,34 | - | 10,34 | 4,31 | - | - | 29,68 | 30,123 |
| III | 1 | - | 0 | 10 | - | 1 | 4 | - | - | 9 | 4 |
| IV | 0 | - | 0 | 6 | - | 0 | 3 | - | - | 1 | 5 |
| Follow-up (months) | 17.35 | 17.40 | 23.87 | 20.20 | 16.46 | 20.86 | 26.78 | 18.93 | 17.28 | 23.89 | 19.57 |
| **Use of each dataset** |  |  |  |  |  |  |  |  |  |  |  |
| Training set | √ | √ | √ | √ | √ | √ | √ |  |  |  |  |
| Testing set |  |  |  |  |  |  |  | √ | √ | √ | √ |

Notes: a: Agilent-014850 Whole Human Genome Microarray 4 × 44K G4112F; b: Affymetrix Human Gene 1.0 ST Array; c: Rosetta/Merck Human RSTA Custom Affymetrix 2.0 microarray; d: Agilent-014850 Whole Human Genome Microarray 4 × 44K G4112F; e: Illumina HiSeq 2000; f: Microarray-based gene expression; g: RNA sequencing (RNA-se1) -based gene expression. NM, not mentioned.

| **Entrez Gene ID** | **Gene Symbol** | **Full name** |
| --- | --- | --- |
| 218 | ALDH3A1 | aldehyde dehydrogenase 3 family member A1 |
| 220 | ALDH1A3 | aldehyde dehydrogenase 1 family member A3 |
| 221 | ALDH3B1 | aldehyde dehydrogenase 3 family member B1 |
| 222 | ALDH3B2 | aldehyde dehydrogenase 3 family member B2 |
| 119391 | GSTO2 | glutathione S-transferase omega 2 |
| 221357 | GSTA5 | glutathione S-transferase alpha 5 |
| 2938 | GSTA1 | glutathione S-transferase alpha 1 |
| 2939 | GSTA2 | glutathione S-transferase alpha 2 |
| 2940 | GSTA3 | glutathione S-transferase alpha 3 |
| 2941 | GSTA4 | glutathione S-transferase alpha 4 |
| 2944 | GSTM1 | glutathione S-transferase mu 1 |
| 2946 | GSTM2 | glutathione S-transferase mu 2 |
| 2947 | GSTM3 | glutathione S-transferase mu 3 |
| 2948 | GSTM4 | glutathione S-transferase mu 4 |
| 2949 | GSTM5 | glutathione S-transferase mu 5 |
| 2950 | GSTP1 | glutathione S-transferase pi 1 |
| 2952 | GSTT1 | glutathione S-transferase theta 1 |
| 2953 | GSTT2 | glutathione S-transferase theta 2 |
| 373156 | GSTK1 | glutathione S-transferase kappa 1 |
| 4257 | MGST1 | microsomal glutathione S-transferase 1 |
| 4258 | MGST2 | microsomal glutathione S-transferase 2 |
| 4259 | MGST3 | microsomal glutathione S-transferase 3 |
| 653689 | GSTT2B | glutathione S-transferase theta 2B |
| 9446 | GSTO1 | glutathione S-transferase omega 1 |
| 124 | ADH1A | alcohol dehydrogenase 1A (class I), alpha polypeptide |
| 125 | ADH1B | alcohol dehydrogenase 1B (class I), beta polypeptide |
| 126 | ADH1C | alcohol dehydrogenase 1C (class I), gamma polypeptide |
| 127 | ADH4 | alcohol dehydrogenase 4 (class II), pi polypeptide |
| 128 | ADH5 | alcohol dehydrogenase 5 (class III), chi polypeptide |
| 130 | ADH6 | alcohol dehydrogenase 6 (class V) |
| 131 | ADH7 | alcohol dehydrogenase 7 (class IV), mu or sigma polypeptide |

**Table S2.** Genes of the 00982_1 subpathway

**Table S3.** Predictive power of published gene signatures compared with path:00982_1 in 11 datasets used in the present study

| **Published signature /path：00982_1** | **No. of**  **genes** | **GSE21501** | **GSE28735** | **GSE57495** | **GSE62452** | **GSE71729** | **GSE78229** | **GSE79668** | **ICGA.AU.array** | **ICGC.AU.seq** | **ICGC.CA.seq** | **TCGA** |
| --- | --- | --- | --- | --- | --- | --- | --- | --- | --- | --- | --- | --- |
| (Birnbaum, et al., 2017) | 23 | 0.722 | 0.487 | 0.125 | 0.128 | 0.064 | 0.171 | ***0.009*** | ***1.60E-05*** | 0.248 | 0.690 | 0.799 |
| (Chen, et al., 2015) | 15 | ***0.049*** | 0.094 | 0.360 | ***0.008*** | 0.189 | ***0.006*** | 0.170 | ***2.20E-05*** | 0.173 | 0.392 | 0.128 |
| (Newhook, et al., 2014) | 12 | ***0.0217*** | 0.502 | 0.648 | 0.136 | 0.346 | 0.734 | 0.391 | 0.697 | 0.454 | 0.313 | ***0.019*** |
| (Haider, et al., 2014) | 36 | ***0.011*** | ***0.004*** | 0.906 | ***3.90E-05*** | 0.109 | ***0.001*** | 0.149 | ***7.00E-06*** | ***5.00E-06*** | 0.135 | 0.268 |
| (Stratford, et al., 2010) | 6 | 0.467 | 0.419 | 0.079 | 0.228 | 0.581 | 0.373 | 0.398 | 0.315 | 0.109 | 0.232 | 0.476 |
| (Wang, et al., 2013) | 27 | 0.595 | 0.576 | 0.843 | 0.219 | 0.744 | 0.182 | 0.707 | ***0.001*** | 0.0507 | ***0.049*** | 0.812 |
| (Kirby, et al., 2016) | 13 | 0.268 | 0.939 | 0.854 | 0.226 | 0.079 | 0.237 | 0.345 | 0.075 | 0.071 | 0.837 | 0.053 |
| (Shi, et al., 2017) | 16 | 0.918 | 0.701 | 0.442 | 0.153 | 0.669 | 0.148 | 0.826 | 0.725 | 0.180 | 0.052 | ***0.001*** |
| (Raman, et al., 2018) | 5 | 0.149 | 0.101 | 0.637 | ***0.010*** | 0.267 | ***0.010*** | 0.826 | ***0.001*** | 0.173 | 0.392 | 0.129 |
| path：00982_1 | 31 | ***0.041*** | ***0.010*** | 0.421 | ***1.70E-04*** | 0.163 | ***9.80E-04*** | 0.571 | ***0.001*** | ***0.008*** | ***0.037*** | 0.111 |

Notes：Bold italic indicates that P-value <0.05.

**Supplementary Figures**

| **Figure S1 Search strategy in GEO database** | |
| --- | --- |
| Number | Strategies |
| #1 | “Carcinoma, Pancreatic Ductal” [Mesh] |
| #2 | Carcinomas, Pancreatic Ductal |
| #3 | Ductal Carcinomas, Pancreatic |
| #4 ~#16 | **…** |
| #17 | Pancreas Duct-Cell Carcinoma |
| #18 | Pancreas Duct-Cell Carcinomas |
| #19 | Pancreatic ductal adenocarcinoma |
| #20 | #1 OR #2 OR #3 OR #4 OR #5 OR #6 OR #7 OR #8 OR #9 OR #10 OR #11 OR #12 OR #13 OR #14 OR #15 OR #16 OR #17 OR #18 OR #19 |
| #21 | “Pancreatic Neoplasms” [Mesh] |
| #22 | Neoplasm, Pancreatic |
| #23 | Pancreatic Neoplasm |
| #24 ~#35 | … |
| #36 | Cancers, Pancreatic |
| #37 | Pancreatic Cancers |
| #38 | Cancer of the Pancreas |
| #39 | #21 OR #22 OR #23 OR #24 OR #25 OR #26 OR #27 OR #28 OR #29 OR #30 OR #31 OR #32 OR #33 OR #34 OR #35 OR #36 OR #37 OR #38 |
| #40 | #20 OR #39 |
| Note: A total of 6573 datasets were extracted by this strategy up to March, 2019. GEO, Gene Expression Omnibus (https://www.ncbi.nlm.nih.gov/geo/). | |

**Figure S**2


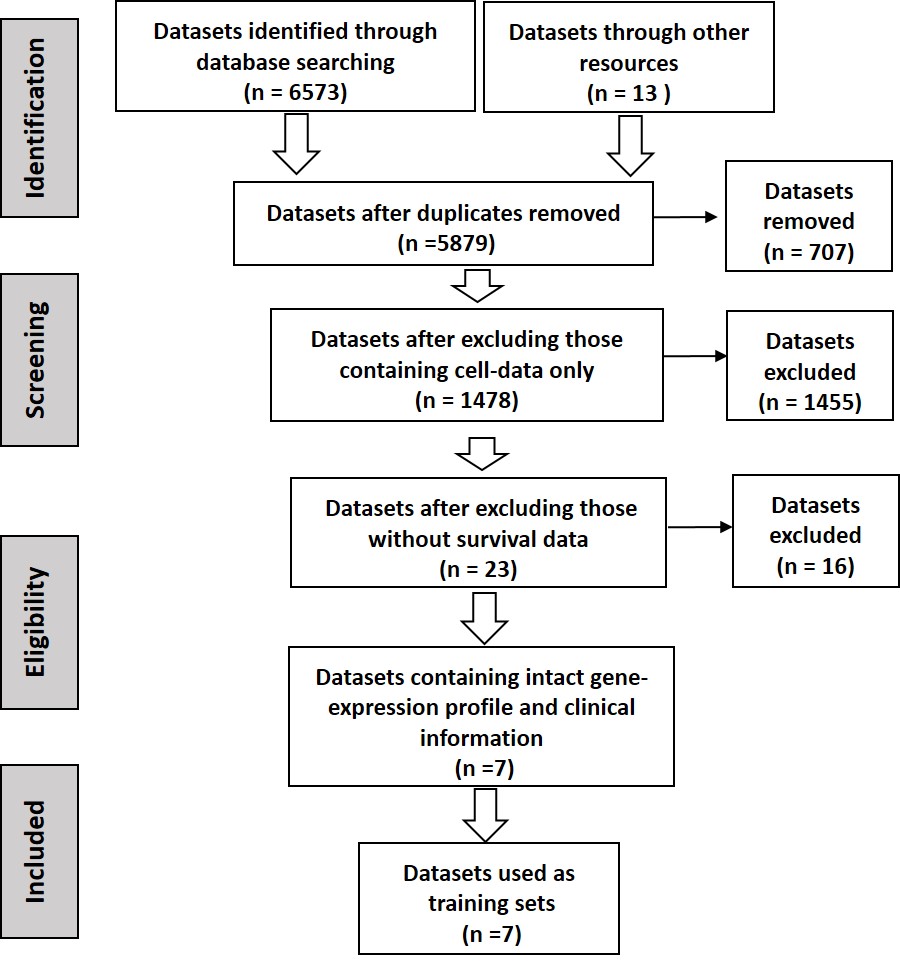


Figure S2. Flow diagram of dataset selection strategies from GEO databases. GEO, Gene Expression Omnibus (https://www.ncbi.nlm.nih.gov/geo/).

**Figure S**3


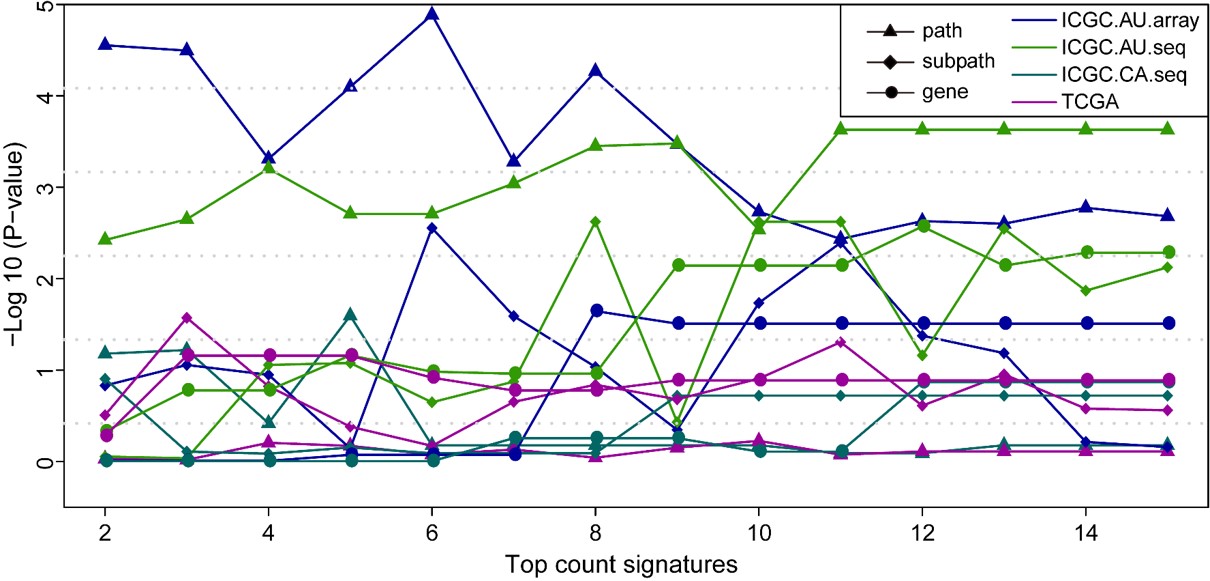


Figure S3. Accumulative predictive abilities of signatures at gene, subpathway and pathway levels. Accumulated top robust signatures (Ranked 2^nd^ -15^th^ identified in Figure 1) are evaluated in 4 testing sets. The prognostic P-value is calculated and -log10 of P-value used as Y-axis.

**Figure S**4


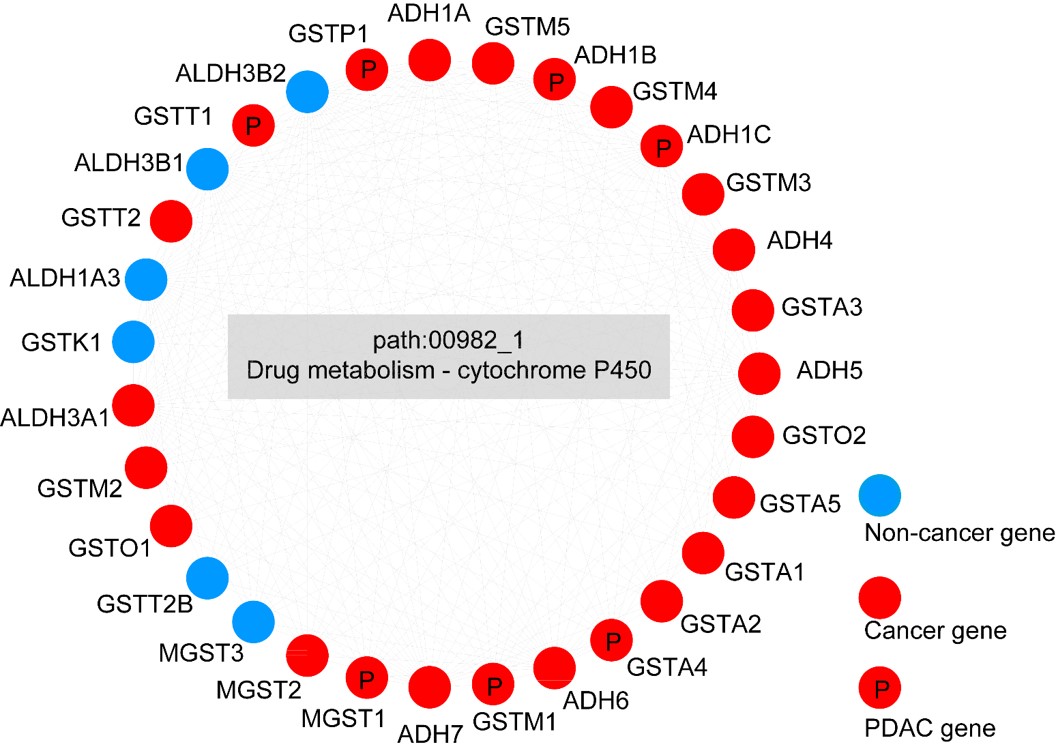


Figure S4. Genes in the path:00982_1 subpathway and their subgrouping according to the Kyoto Encyclopedia of Genes and Genomes (KEGG) (Kanehisa and Goto, 2000).

**Figure S**5


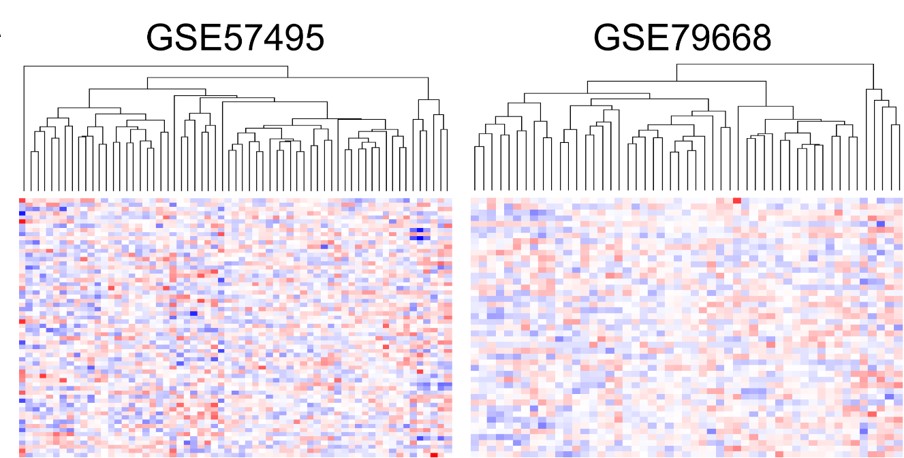


Figure S5. The Collision classification (Collisson, et al., 2011) is not applicable for GSE57495 and GSE79668 datasets.

**Figure S**6


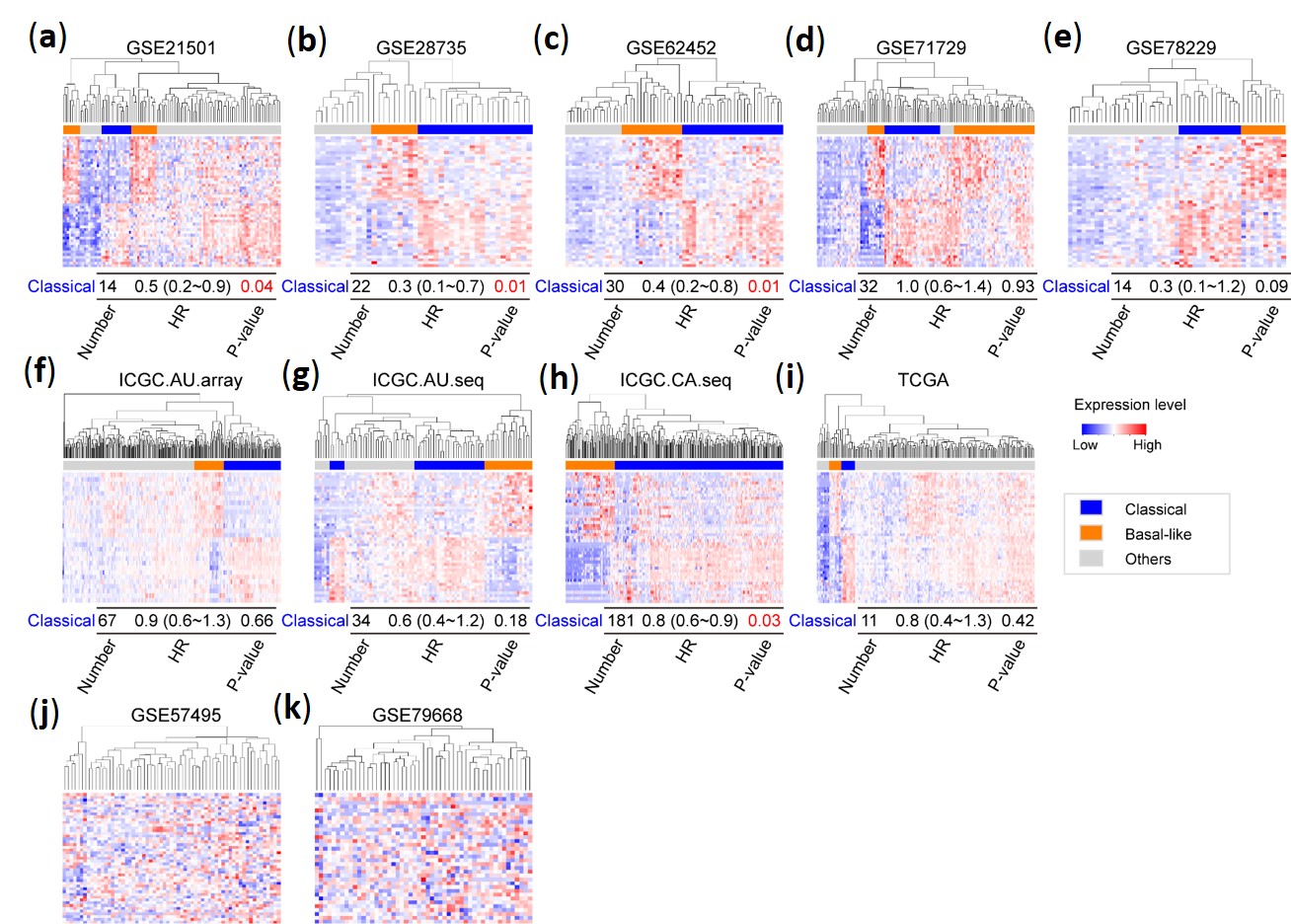


Figure S6. The prognostic capacity of Path:00982_1 signature in PDAC classical subtype. Patients in each dataset are stratified into classical, basal-like and others subtypes by using a classification by Moffitt with modification (Moffitt, et al., 2015). Hazard ratio (HR) and P-value for each subtype are calculated. Data from 7 training datasets are shown in (**a**-**e**) and 4 testing datasets in (**f**-**i**). (**j**, **k**) This classification is not applicable to GSE57495 and GSE79668 datasets. The red-colored number indicates that the path:00982_1 signature has a significant predictive ability.

**Figure S**7


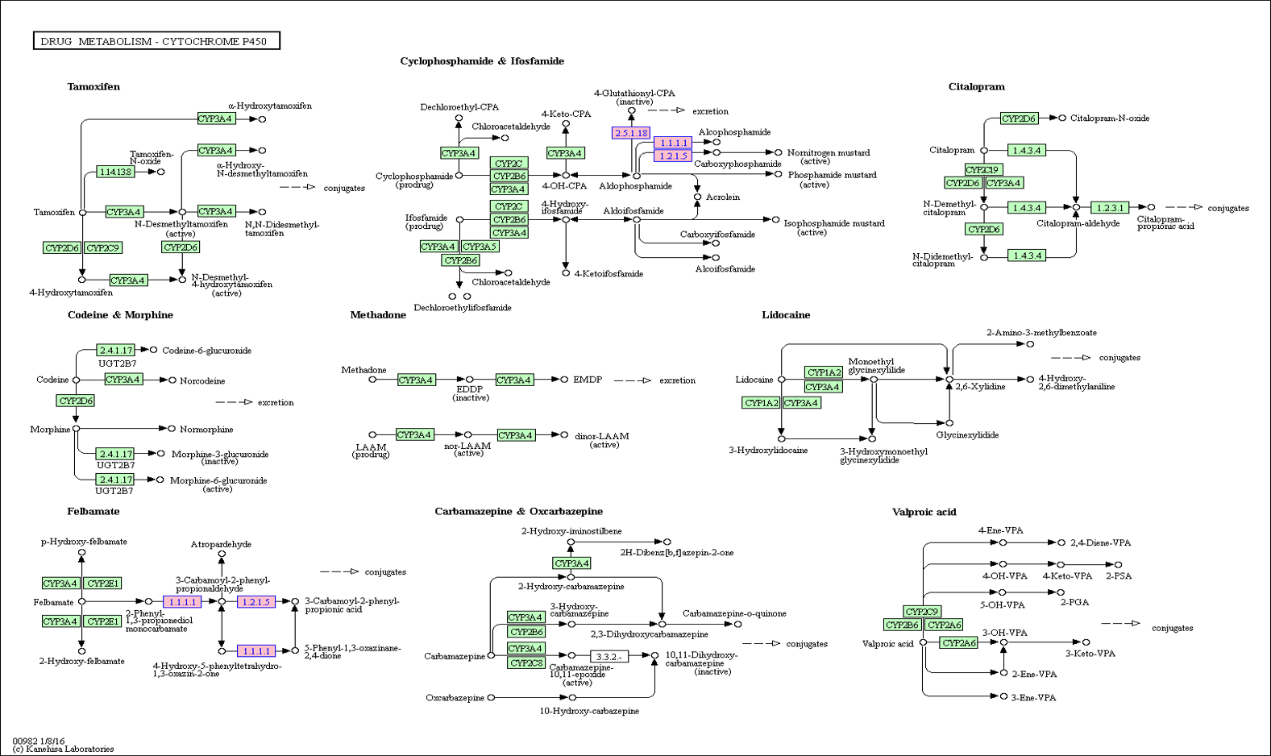


Figure S7. The association of path:00982_1 subpathway with other pathways and its location in the whole path:00982 pathway. Image quoted from the Kyoto Encyclopedia of Genes and Genomes (KEGG) database (Kanehisa and Goto, 2000). The path:00982_1 subpathway indicated by pink color consists of three enzyme complexes (EC number: 2.5.1.18 [glutathione transferase; glutathione S-transferase; glutathione S-alkyltransferase; glutathione S-aryltransferase; S-(hydroxyalkyl)glutathione lyase; glutathione S-aralkyltransferase; glutathione S-alkyl transferase], 1.1.1.1 [alcohol dehydrogenase; aldehyde reductase; ADH; alcohol dehydrogenase (NAD);aliphatic alcohol dehydrogenase; ethanol dehydrogenase; NAD-dependent alcohol dehydrogenase; NAD-specific aromatic alcohol dehydrogenase; NADH-alcohol dehydrogenase; NADH-aldehyde dehydrogenase; primary alcohol dehydrogenase; yeast alcohol dehydrogenase] and 1.2.1.5[aldehyde dehydrogenase; ALDH]).

**References**

Birnbaum, D.J.*, et al.* A 25-gene classifier predicts overall survival in resectable pancreatic cancer. *BMC medicine* 2017;15(1):170-184.

Chen, D.-T.*, et al.* Prognostic fifteen-gene signature for early stage pancreatic ductal adenocarcinoma. *PloS one* 2015;10(8):e0133562.

Collisson, E.A.*, et al.* Subtypes of pancreatic ductal adenocarcinoma and their differing responses to therapy. *Nature medicine* 2011;17(4):500-503.

Haider, S.*, et al.* A multi-gene signature predicts outcome in patients with pancreatic ductal adenocarcinoma. *Genome medicine* 2014;6(12):105-116.

Kanehisa, M. and Goto, S. KEGG: kyoto encyclopedia of genes and genomes. *Nucleic acids research* 2000;28(1):27-30.

Kirby, M.K.*, et al.* RNA sequencing of pancreatic adenocarcinoma tumors yields novel expression patterns associated with long-term survival and reveals a role for ANGPTL4. *Molecular oncology* 2016;10(8):1169-1182.

Moffitt, R.A.*, et al.* Virtual microdissection identifies distinct tumor-and stroma-specific subtypes of pancreatic ductal adenocarcinoma. *Nature genetics* 2015;47(10):1168-1191.

Newhook, T.E.*, et al.* A thirteen-gene expression signature predicts survival of patients with pancreatic cancer and identifies new genes of interest. *PloS one* 2014;9(9):e105631.

Raman, P.*, et al.* Pancreatic cancer survival analysis defines a signature that predicts outcome. *PloS one* 2018;13(8):e0201751.

Shi, G.*, et al.* A novel messenger RNA signature as a prognostic biomarker for predicting relapse in pancreatic ductal adenocarcinoma. *Oncotarget* 2017;8(67):110849-110860.

Stratford, J.K.*, et al.* A six-gene signature predicts survival of patients with localized pancreatic ductal adenocarcinoma. *PLoS medicine* 2010;7(7):e1000307.

Wang, W.Y.*, et al.* A gene expression signature of epithelial tubulogenesis and a role for ASPM in pancreatic tumor progression. *Gastroenterology* 2013;145(5):1110-1120.
